# Beyond vulnerability: reflective selfhood, rural woman identity reinterpretation, and agency under structural constraint

**DOI:** 10.3389/fpsyg.2026.1893046

**Published:** 2026-08-14

**Authors:** Wenyuan Yang, Xiaomin Zhu

**Affiliations:** 1 School of Law and Public Administration, Jia Ying University, Meizhou, China; 2 Social Development College of East China University of Political Science and Law, Shanghai, China

**Keywords:** adversity, agency, narrative identity, reflective selfhood, resilience, rural woman identity reinterpretation, rural women

## Abstract

Personality, social, and narrative psychology increasingly call for context-sensitive approaches to understanding how selfhood and agency are organized under structurally unequal conditions. This three-wave field survey examined prospective associations among adversity experiences, reflective selfhood, rural woman identity reinterpretation, family decision-making power, psychological resilience, and agency among rural women in China. Reflective selfhood and rural woman identity reinterpretation were conceptualized as preliminary, narrative-informed, context-sensitive operationalizations of the interpretive processes through which women make sense of lived adversity, social position, responsibility, dignity, and possible agency. Analyses included measurement validation, baseline-adjusted lagged models, attrition robustness checks, and Monte Carlo tests of indirect associations. T1 adversity experiences were positively associated with T2 reflective selfhood and T2 rural woman identity reinterpretation. In turn, both T2 processes were positively associated with T3 psychological resilience and agency after baseline controls were included. Bootstrapped indirect associations through both T2 processes were positive. Exploratory moderation results suggested that family decision-making power may shape the association between reflective selfhood and agency, although this interaction should be interpreted cautiously because *post hoc* power was limited. The three-wave design strengthens temporal ordering, but the findings should be interpreted as self-report-based prospective associations rather than causal evidence. The study contributes to narrative-informed personality and social psychology by proposing and preliminarily testing a context-sensitive model of rural women’s selfhood and agency under structural constraint, while avoiding the implication that hardship itself is beneficial.

## 1 Introduction

Scholars in personality and social psychology have long examined selfhood, identity, motivation, agency, and adaptation, yet many influential theories in these fields have been developed primarily from urban, educated, and relatively advantaged samples. This creates a persistent problem for psychological science: theories of self and resilience may appear universal while being grounded in social worlds where personal choice, educational mobility, and institutional support are more readily available. Rural women’s lives offer a critical site for rethinking this assumption because their psychological development is shaped not only by individual traits, but also by gendered family obligations, care work, agricultural and local labor, limited services, mobility constraints, and rural–urban inequality. In everyday life, these conditions may appear in decisions about children’s schooling after a poor harvest, elder care when husbands or sons migrate for work, household expenses without final authority over money, or the social consequences of visible family conflict within village life. A context-sensitive psychology must therefore ask not only whether rural women are distressed or resilient, but also how they interpret their life conditions and what forms of agency become possible within them. Rural women occupy intersecting positions in family, community, labor, and gender systems. They often carry responsibility for children, older adults, household coordination, agricultural work, and emotional maintenance within families, while also facing restricted access to education, formal employment, health resources, and decision-making authority. Such conditions may generate chronic adversity rather than a single traumatic event. The adversity of rural women is often ordinary and repeated: a postponed training opportunity, an unrecognized contribution to farm or family businesses, the expectation to prioritize sons’ education or elder care, the obligation to maintain harmony in marital and kinship relations, limited voice in household decisions, and the social expectation that endurance is a virtue. These experiences are psychological, but they are also social and structural (Bandura, 2006; Deci and Ryan, 2000).

A narrative psychology perspective helps clarify why these conditions matter for selfhood and agency. Psychology should not ask only whether rural women are vulnerable, resilient, or agentic. It should also ask how they organize lived experience narratively, how they interpret family obligation, care responsibility, low recognition, rural–urban inequality, and limited choice, and how these interpretations become part of self-understanding and possible action. Narrative identity theory emphasizes that people make sense of their lives by linking events, social positions, values, and imagined futures into accounts of who they are and what they can do (Bruner, 1990; McAdams and McLean, 2013). For rural women, this narrative work is not an abstract autobiographical exercise. It occurs in the everyday language of duty, endurance, dignity, family responsibility, community knowledge, and constrained possibility. Concepts such as autobiographical meaning-making, self-authorship, meaning reconstruction, and narrative agency are therefore useful for understanding how women interpret structural constraint without denying its material reality. This study uses a three-wave quantitative design to examine measurable narrative-interpretive processes rather than full life stories. Much existing literature has treated rural women primarily as recipients of disadvantage or as a psychologically vulnerable population. This focus remains important because poverty, discrimination, care burden, and gendered power inequalities can undermine well-being. However, a vulnerability-only framing can flatten rural women’s subjectivity. It may describe what happens to them without explaining how they interpret what happens, how they reconstruct identity, or how they develop forms of resilience and agency under constraint. The present study addresses this gap through a burden-and-response framing. Adversity is treated first as a structural burden that may undermine psychological functioning. At the same time, some women may respond to long-term constraint by developing reflective and identity-based resources that partially buffer harm. The positive indirect associations tested in this paper should therefore be interpreted as adaptive responses to constraint, not as evidence that adversity itself has developmental value (Crossley, 2000; Singer, 2004).

Reflective selfhood is central to this argument. It refers to the capacity to interpret one’s experiences, social position, constraints, and choices in ways that support self-understanding and future-oriented agency. Reflection is not equivalent to rumination. Rumination may trap individuals in repetitive negative thought, whereas reflective selfhood involves making sense of experience and locating oneself within wider social conditions. For rural women, reflective selfhood may involve asking why certain sacrifices have been expected, why unpaid care or agricultural labor is treated as natural rather than skilled, what kinds of contribution have been overlooked, and what possibilities remain open despite constraint. It provides a bridge between endurance and agency. Conceptually, reflective selfhood differs from general self-reflection because it is explicitly tied to social position and lived constraint; it differs from rumination because it organizes experience rather than repeatedly dwelling on distress; it differs from self-efficacy because it concerns interpretation and authorship of life experience rather than confidence in task performance; and it differs from resilience because it is a meaning-making process rather than an adaptive outcome. Rural woman identity reinterpretation is the second mechanism. Rural womanhood may be socially marked by stereotypes of backwardness, dependence, limited education, or domestic obligation. Yet identity is not only imposed; it can also be reinterpreted. The term reinterpretation is used deliberately but cautiously. It refers not merely to positive identity evaluation or rural pride, but to the reinterpretation of a socially devalued identity after experiences of undervaluation, stigma, or low recognition. Rural women may come to understand their identities as sources of knowledge, competence, responsibility, and community contribution: knowing how to coordinate seasonal work, care for children and elders, preserve kinship ties, manage scarce resources, and maintain community continuity. Such reinterpretation does not deny inequality. Rather, it reframes an undervalued position as a site of dignity and collective meaning. Because the current data did not include a separate identity-devaluation scale, the construct should be understood as positive rural woman identity reinterpretation rather than a complete pre-post reconstruction process. Finally, the translation of reflection into agency may depend on context. Reflective self-understanding may have limited behavioral consequences when women have little voice in household decisions. Family decision-making power is therefore treated as relational space for possible agency. Given the modest size of the interaction and limited *post hoc* power, however, this part of the model is framed as exploratory rather than as the central contribution of the study. This study contributes by proposing and preliminarily testing a narrative-informed, context-sensitive model of rural women’s selfhood and agency under structural constraint. Its central contribution lies in the theoretical and empirical positioning of reflective selfhood and rural woman identity reinterpretation as narrative-informed interpretive processes. The study also examines family decision-making power as a possible relational boundary condition, while treating the moderation finding as preliminary evidence that requires replication in larger samples (Archer, 2007; Giddens, 1991).

## 2 Literature review and hypothesis development

### 2.1 Rural women’s adversity experiences in context

Adversity experiences among rural women should be understood as chronic, relational, and structurally embedded. They include economic pressure, care burden, restricted educational opportunity, low recognition of labor, pressure from marriage and kinship norms, limited voice in household decisions, and rural–urban resource inequality. These experiences are not merely external stressors. They also become life materials that women may need to interpret, narrate, and integrate into their understanding of self, responsibility, dignity, and possible action. This interpretive demand does not mean that adversity is beneficial in itself. Rather, it suggests that structural burden can become part of the psychological work through which women make sense of constrained lives (Bourdieu, 1990; Kabeer, 1999).

This argument requires careful qualification. The study does not claim that adversity is beneficial, nor does it romanticize hardship as a source of growth, especially when such hardship reflects preventable inequality. Instead, the argument is that women may develop interpretive responses while living under constraint. Adversity remains a structural burden, while reflective selfhood and rural woman identity reinterpretation are treated as possible narrative responses to that burden (Bonanno, 2021; Hobfoll, 1989).

*H1*: Adversity experiences will be positively associated with reflective selfhood among rural women, consistent with the idea that repeated constraint may require interpretive self-organization.

### 2.2 Reflective selfhood as a meaning-making mechanism

Reflective selfhood refers to more than private introspection. Drawing on McAdams’s actor, agent, and author distinction, it is treated here as an author level narrative interpretive process through which women organize biography, social position, responsibility, and future possibility under structural constraint (McAdams, 2013; McAdams and McLean, 2013). Reflective selfhood is not a complete life story and is not identical to general self-reflection. It is a narrower, measurable process through which women interpret how past hardship, present obligations, and possible futures belong to the same self. In this sense, narrative identity is the broader life story framework, while reflective selfhood is the study’s context sensitive operationalization of self-interpretation under constraint.

Reflective selfhood is therefore expected to show positive prospective associations with later psychological resilience and agency. In the three wave design, adversity and family decision-making power are positioned at T1, reflective selfhood and rural woman identity reinterpretation at T2, and resilience and agency at T3. This separation provides a stronger temporal basis for prospective indirect associations than a two wave design, although it still does not support causal inference. The mechanism focused interpretation is not that adversity automatically strengthens resilience. Instead, adversity may become psychologically consequential through the interpretive work by which women make sense of it (Lazarus and Folkman, 1984; Luthar et al., 2000).

*H2a*: Reflective selfhood will be positively associated with psychological resilience.

*H2b*: Reflective selfhood will be positively associated with agency.

*H3a*: Adversity experiences will show a positive indirect association with psychological resilience through reflective selfhood.

*H3b*: Adversity experiences will show a positive indirect association with agency through reflective selfhood.

### 2.3 Rural woman identity reinterpretation among rural women

Identity is not a fixed label. It is formed through social recognition, comparison, collective meaning, and personal reinterpretation. Rural women may encounter devaluing labels associated with rural origin, limited schooling, domestic obligation, or economic dependence. Rural woman identity reinterpretation is therefore conceptualized as the narrative revaluation of a socially devalued identity. It is not simple pride and not blind positivity. Rather, it captures the extent to which rural womanhood is re described as knowledge, competence, care contribution, dignity, responsibility, and community continuity (Tajfel and Turner, 1979; Turner et al., 1987).

This process is expected to support psychological resilience because it strengthens dignity, belonging, and continuity. It is also expected to support agency because women who reinterpret their social role as meaningful may feel more entitled to speak, decide, and participate. This argument remains cautious. Because the data do not include a separate identity devaluation scale or a repeated measure of negative identity meaning, the measure captures positive identity reinterpretation rather than a full reconstruction trajectory from devaluation to revaluation (Jetten et al., 2012; Sun, 2025).

*H4a*: Adversity experiences will be positively associated with rural woman identity reinterpretation.

*H4b*: rural woman identity reinterpretation will be positively associated with psychological resilience.

*H4c*: rural woman identity reinterpretation will be positively associated with agency.

*H5a*: Adversity experiences will show a positive indirect association with psychological resilience through rural woman identity reinterpretation.

*H5b*: Adversity experiences will show a positive indirect association with agency through rural woman identity reinterpretation.

### 2.4 Family decision-making power as a contextual moderator

The household is one of the most immediate social structures in which rural women’s agency is enabled or constrained. A woman may reflect deeply on her life and reinterpret her identity, yet still lack the relational space to act if her voice is ignored in decisions about money, work, care, mobility, health, or children’s education. Family decision-making power is therefore examined as a possible relational condition under which reflective selfhood is more strongly associated with agency. Because the expected interaction is small, the moderation analysis is treated as exploratory (Li, 2022).

Exploratory moderation reasoning suggests that when family decision-making power is high, reflective selfhood may be more strongly associated with agency because reflection has more room to become choices, negotiation, and participation. When decision-making power is low, reflection may still support inner clarity, but its behavioral expression may be constrained. This expectation requires replication in larger samples (Jacka, 2012; Zhang, 2013).

*H6a*: Family decision-making power will show an exploratory moderating association with the reflective selfhood to agency path, such that the association is expected to be stronger when decision-making power is higher.

*H6b*: Family decision-making power will show an exploratory moderated indirect association for the adversity to agency pathway through reflective selfhood.

### 2.5 Narrative and interpretive scope of the model

Although this study does not use qualitative narrative interviews, it is narrative informed because it operationalizes measurable narrative interpretive processes within a three wave psychological model. The unit of analysis is not full life narratives, life history accounts, interview stories, or discourse sequences. Rather, the unit of analysis is the set of reflective and identity based processes through which women organize adversity, social position, responsibility, dignity, and possible agency. Reflective selfhood captures how women interpret biography and future possibility under constraint. Rural woman identity reinterpretation captures how women revalue a socially devalued identity as knowledge, competence, care contribution, and community continuity. This positioning is important for the scope of the article. The study contributes to narrative psychology not by analyzing stories in women’s own words, but by testing whether narrative inspired self and identity processes are prospectively associated with resilience and agency in a three wave field survey. The approach is therefore best described as narrative informed quantitative psychology (Adler et al., 2016; Hammack, 2008).

### 2.6 Scope conditions and falsifiability

The model is not intended to imply that adversity routinely predicts growth, nor that reflective interpretation can compensate for structural inequality. Its scope is narrower. The proposed process should be most relevant in settings where women face repeated but narratable constraints, maintain some relational connection to family or community life, and have at least minimal opportunities to compare, reinterpret, and discuss their experiences. It should be less applicable in contexts of acute crisis, coercive violence, severe social isolation, or conditions where women have almost no room to act on reflection. In such cases, adversity may overwhelm meaning making processes rather than become material for reflective selfhood (Tedeschi and Calhoun, 2004; Ungar, 2011).

The model is also falsifiable. If adversity were unrelated or negatively related to reflective selfhood after baseline controls, the argument that repeated constraint can stimulate interpretive self-understanding would be weakened. If reflective selfhood showed no association with resilience or agency once identity reinterpretation and prior outcomes were controlled, it would suggest that the construct adds little beyond adjacent positive self-processes. If family decision-making power did not condition the reflection-to-agency link, the claim that agency requires relational space would need to be revised. These boundaries are important because the study aims to offer a cautious context sensitive account, not a generalized claim that hardship is psychologically productive.

## 3 Methods

### 3.1 Research design and participants

This study used a three wave field survey to examine adversity experiences, reflective selfhood, rural woman identity reinterpretation, psychological resilience, and agency among rural women. T1 measured adversity experiences, family decision-making power, baseline psychological variables, and demographic controls. T2, approximately 4 to 6 weeks after baseline, measured reflective selfhood and rural woman identity reinterpretation. T3, approximately 8–12 weeks after baseline, measured psychological resilience and agency. These intervals were chosen to be long enough for intervening interpretive processes to be temporally separated from baseline adversity and short enough to reduce panel loss in rural communities where seasonal work, caregiving, and migration can disrupt follow up. The design supports temporally ordered prospective associations, although it does not permit strong causal inference (Podsakoff et al., 2012).

Participants were recruited through village and township communities in rural China. Recruitment was coordinated with local women’s federation contacts, township service workers, and village level community liaisons, but participation was explained as voluntary and independent of access to public services, community benefits, or village administration. The inclusion criteria were: identifying as a woman, being at least 18 years old, having lived in a rural community for at least 3 years, and being able to complete the questionnaire independently or with standardized reading support. The initial sample included 524 rural women aged 18–65. At T2, 458 participants provided usable responses, and at T3, 411 participants provided usable outcome responses.

Fieldwork used a mixed paper-and-pencil and assisted-questionnaire procedure. Trained female fieldworkers administered surveys in private or semi-private community settings and were instructed to read items aloud neutrally for participants with lower literacy, visual difficulty, or dialect-related comprehension needs. Fieldworkers were trained not to explain items in leading ways, not to suggest socially desirable answers, and not to allow family members, village cadres, or community staff to view responses. Anonymous matching codes were used to link T1, T2, and T3 surveys without storing names in the analysis file. Attrition was handled by documenting follow-up status, comparing retained and dropped participants on T1 variables, and estimating inverse probability weighted robustness models (Brislin, 1980).

The study protocol was reviewed and approved before data collection through the author’s institutional ethics review process. Participants provided written informed consent or, where literacy barriers were present, orally witnessed informed consent using an approved script. They were told that they could skip sensitive questions or withdraw at any wave without penalty. Questionnaires did not collect names in the analytic dataset; contact information used for follow up was stored separately from survey responses and deleted after matching. Participants who reported distress or asked for help were given referral information for local public health, women’s federation, or social service contacts.

### 3.2 Measures

Adversity experiences were measured with 10 items designed to capture economic hardship, care burden, restricted opportunity, low recognition of labor, gendered family expectations, and rural urban resource inequality. The item wording intentionally avoided treating adversity as a single traumatic event. Instead, it reflected repeated pressures such as sacrificing personal needs for family responsibilities, having fewer education or training opportunities, carrying invisible household responsibilities, and feeling excluded from resources more available to urban women. Reflective selfhood was measured with six items developed from self-reflection, meaning making, and narrative identity traditions. Rural woman identity reinterpretation was measured with six items capturing positive reinterpretation of rural womanhood, dignity, contribution, and collective value. Psychological resilience was measured with 10 items adapted from brief resilience traditions and CD-RISC style content. Agency was measured with six items adapted from personal agency and self-efficacy traditions. Family decision-making power was measured with six items assessing voice, respect, and participation in household decisions (Connor and Davidson, 2003; Campbell-Sills and Stein, 2007).

All items used five-point response scales. Higher scores indicated higher levels of the relevant construct. Scale scores were computed as item means. Control variables included age, education, marital status, employment status, household income, number of children, region, years living in a rural community, and migration experience.

### 3.3 Scale development and construct validation

Because reflective selfhood and rural woman identity reinterpretation are preliminary, context-sensitive operationalizations, item development followed a conservative and transparent scale-development process. Items were derived from theories of reflexivity, meaning-making, narrative identity, social identity, collective dignity, and rural gendered labor. The present study should therefore be read, in part, as an initial effort to operationalize reflective selfhood and rural woman identity reinterpretation in the context of rural women’s lives. Reflective selfhood items focused on the interpretation of life experience and social position rather than on confidence, optimism, or emotional recovery. Rural woman identity reinterpretation items focused on reinterpreting rural womanhood as knowledge, contribution, dignity, and collective value rather than on simply reporting rural pride. A small panel of experts in rural sociology, gender studies, and social psychology reviewed the item pool. Cognitive pretesting with rural women was then used to evaluate wording clarity, sensitivity, and comprehension. Items were revised to reduce abstraction, avoid stigmatizing language, and support understanding among respondents with different educational backgrounds. Translation, back-translation, and plain-language adjustment were used where needed. These measures should therefore be treated as initial operationalizations that require further validation rather than as established scales (Nussbaum, 2011; Sen, 1999).

The construct boundary of reflective selfhood was treated as a central validation issue. Reflective selfhood is defined here as a socially situated, author-level process through which women interpret biography, constraint, responsibility, and future possibility. It is related to narrative identity, but it is narrower than a full life story and was measured as a structured self-interpretive orientation rather than as an interview-based narrative. The construct should therefore be interpreted as a preliminary measurable proxy for narrative self-interpretation, not as a mature narrative identity scale. Reflective selfhood is needed beyond existing constructs because the central theoretical problem is not simply whether rural women feel competent, recover from stress, or hold positive self-beliefs. Rather, the issue is how women interpret the relationship between personal biography and socially patterned constraint, and how that interpretation makes future possibility psychologically thinkable without denying inequality. Rural woman identity reinterpretation was also treated cautiously. Because the data do not include a separate identity-devaluation or stigma scale, the measure should be interpreted as positive rural woman identity reinterpretation rather than as definitive evidence of a full identity-reconstruction trajectory. The construct captures the revaluation of rural womanhood as meaningful, competent, and socially contributive. It should therefore be treated as an initial, context-sensitive measure requiring additional validation across regions, ethnic groups, age cohorts, and family structures. Discriminant validity between reflective selfhood and rural woman identity reinterpretation was examined both conceptually and empirically. Reflective selfhood concerns the author-level interpretation of one’s biography and social position, consistent with McAdams’s author concept. Rural woman identity reinterpretation concerns the revaluation of a socially marked identity. The two constructs were therefore expected to be related but distinct (McAdams, 2013; McAdams and McLean, 2013).

Convergent and discriminant evidence is reported. Reflective selfhood showed a moderate association with a meaning-in-life criterion and a low positive association with brooding rumination, supporting the claim that reflective selfhood is adjacent to, but distinguishable from, rumination. Rural woman identity reinterpretation showed a moderate association with an external rural woman identity criterion. Short-form retest correlations also provided preliminary evidence of stability (Pals, 2006; Polkinghorne, 1988).

### 3.4 Data analysis

Analyses were conducted using Python and semopy. Missingness and attrition were examined by comparing retained and dropped participants on T1 variables. Because attrition was expected to be selective, a retention propensity model was estimated and inverse probability weighting was used as a robustness check. Reliability was assessed using Cronbach’s alpha, composite reliability, and AVE. Because several measures were preliminary context sensitive operationalizations, AVE values near 0.50 were interpreted as evidence of moderate rather than strong convergent validity when composite reliability remained acceptable. Confirmatory factor analysis compared the focal measurement model with merged factor and one factor alternatives. Discriminant validity was evaluated using HTMT and Fornell-Larcker evidence. The structural model estimated baseline-adjusted prospective associations, indirect associations, and exploratory moderation by family decision-making power (Chen, 2007; Hair et al., 2022).

*Post hoc* power was evaluated for the focal interaction term. With the T3 analytic sample (*n* = 411) and approximately 16 predictors, the approximate power to detect a standardized interaction of beta = 0.084 at alpha = 0.05 was 0.40. Approximately 1,107 cases would be required to reach 0.80 power for an effect of this size under the same assumptions. The interaction result is therefore reported as preliminary and exploratory evidence. It should be interpreted cautiously and requires replication in larger samples.

Prespecified interpretation criteria were used to avoid over-reading statistically significant results. Measurement fit was treated as acceptable when CFI and TLI were at or above 0.90 and RMSEA and SRMR were below 0.08. HTMT values below 0.85 were treated as preferred evidence of discriminant validity, and AVE values near 0.50 were interpreted cautiously when composite reliability remained acceptable. Indirect associations were considered supported only when the Monte Carlo 95% interval excluded zero. Because the design separated T1 adversity and family decision-making power, T2 reflective and identity processes, and T3 outcomes, the indirect paths have temporal ordering; nevertheless, the non-experimental design means they should be interpreted as prospective indirect associations rather than definitive prospective indirect association (Muthén and Muthén, 2017; Preacher and Hayes, 2008).

## 4 Results

The study began with 524 participants, retained 458 at T2, and retained 411 at T3 (Table 1). Attrition was moderate and not completely random (Table 2). Participants who dropped out by T3 tended to report slightly higher adversity, lower family decision-making power, lower baseline psychological resilience, and lower baseline agency. The resilience and agency differences were statistically significant, suggesting that the retained panel may underrepresent women facing greater psychological and relational challenges. The IPW adjusted model in Table 3 was therefore used to examine whether the focal moderation estimate was sensitive to selective retention, but this robustness check cannot remove all possible attrition bias.

**Table 1:** Demographic characteristics and retention.

Variable	Category	N/Mean	%/SD
Age	Mean (SD)	42.15	10.72
Education	College or above	56	10.69
Education	Junior middle	223	42.56
Education	Primary or below	118	22.52
Education	Senior/vocational	127	24.24
Marital status	Married	401	76.53
Marital status	Unmarried	57	10.88
Marital status	Widowed/divorced	66	12.60
Employment	Agricultural work	203	38.74
Employment	Local nonfarm work	139	26.53
Employment	Migrant/seasonal work	75	14.31
Employment	Unpaid care work	107	20.42
Region	Central	144	27.48
Region	Eastern	105	20.04
Region	Southwestern	116	22.14
Region	Western	159	30.34
Number of children	Mean (SD)	1.53	1.19
Annual household income	Mean (SD)	10920.52	6142.12
Years living in rural community	Mean (SD)	27.70	12.14
Migration experience	Yes	240	45.80
T1 completed	Count	524	100
T2 retained	Count	458	87.40
T3 retained	Count	411	78.44

**Table 2:** Attrition analysis.

Variable	Retained M	Dropped M	Difference	Cohen d	*p*
Age	42.27	41.72	0.544	0.051	0.644
education_num	1.24	1.21	0.024	0.026	0.813
Adversity experiences	2.88	3.04	−0.159	−0.184	0.098
Family decision-making power	3.05	2.90	0.149	0.170	0.136
Reflective selfhood baseline	3.09	3.01	0.079	0.090	0.428
Psychological resilience baseline	3.04	2.77	0.263	0.302	0.003
Agency baseline	3.09	2.86	0.233	0.268	0.010

**Table 3:** Moderation and robustness checks.

Specification	*N*	Key term	Beta	SE	*p*	Result
Main model with controls	411	RSxFDMP	0.107	0.039	0.006	Robust
Without demographic controls	411	RSxFDMP	0.106	0.038	0.005	Robust
Resilience outcome model	411	z_RS_T2	0.235	0.047	<0.001	Robust
Education-adjusted subsample	411	RSxFDMP	0.106	0.038	0.005	Robust
Marker-variable adjusted model	411	RSxFDMP	0.106	0.039	0.006	Robust
IPW adjusted attrition model	411	RSxFDMP	0.104	0.039	0.007	Robust

Reliability values were acceptable for all focal constructs. Several AVE values were close to or below 0.50, including adversity experiences, psychological resilience, and family decision-making power. This pattern suggests acceptable internal consistency but only moderate convergent validity for some context sensitive constructs (Table 4). The findings should therefore be interpreted with measurement caution, and future studies should refine these scales and test their performance across broader rural populations (Tables 5, 6). The correlation matrix showed a nuanced pattern: adversity was positively associated with reflective selfhood and rural woman identity reinterpretation, but negatively associated with resilience and agency. Reflective selfhood and rural woman identity reinterpretation were positively associated with both outcomes (Table 7).

**Table 4:** Descriptive statistics, reliability, CR, and AVE.

No.	Variable	*N*	M	SD	Alpha	CR	AVE
1.00	Adversity experiences	524	2.91	0.866	0.875	0.899	0.472
2.00	Reflective selfhood	458	3.03	0.866	0.800	0.857	0.501
3.00	Rural woman identity reinterpretation	458	2.99	0.888	0.822	0.871	0.530
4.00	Psychological resilience	411	3.05	0.899	0.884	0.906	0.491
5.00	Agency	411	3.08	0.953	0.843	0.884	0.562
6.00	Family decision-making power	524	3.02	0.875	0.795	0.854	0.496

AVE values at or below 0.50 were observed for Adversity experiences, Psychological resilience, and Family decision-making power. Composite reliability was acceptable, but AVE values near 0.50 indicate only moderate convergent validity for several context sensitive constructs. These preliminary measures should be interpreted with measurement caution and refined in future studies (Table 4).

**Table 5:** HTMT discriminant validity.

Construct pair	HTMT	Criterion	Decision
Adversity experiences/reflective selfhood	0.306	<0.85 preferred	Passed
Adversity experiences/rural woman identity reinterpretation	0.377	<0.85 preferred	Passed
Adversity experiences/psychological resilience	0.108	<0.85 preferred	Passed
Adversity experiences/agency	0.105	<0.85 preferred	Passed
Adversity experiences/family decision-making power	0.436	<0.85 preferred	Passed
Reflective selfhood/rural woman identity reinterpretation	0.470	<0.85 preferred	Passed
Reflective selfhood/psychological resilience	0.397	<0.85 preferred	Passed
Reflective selfhood/agency	0.403	<0.85 preferred	Passed
Reflective selfhood/family decision-making power	0.102	<0.85 preferred	Passed
Rural woman identity reinterpretation/psychological resilience	0.394	<0.85 preferred	Passed
Rural woman identity reinterpretation/agency	0.485	<0.85 preferred	Passed
Rural woman identity reinterpretation/family decision-making power	0.090	<0.85 preferred	Passed
Psychological resilience/agency	0.419	<0.85 preferred	Passed
Psychological resilience/family decision-making power	0.284	<0.85 preferred	Passed
Agency/family decision-making power	0.334	<0.85 preferred	Passed

**Table 6:** Fornell-Larcker matrix.

Construct	Adversity experiences	Reflective selfhood	Rural woman identity reinterpretation	Psychological resilience	Agency	Family decision-making power
Adversity experiences	0.687	0.256	0.319	−0.084	−0.044	−0.363
Reflective selfhood	0.256	0.708	0.381	0.333	0.330	0.043
Rural woman identity reinterpretation	0.319	0.381	0.728	0.336	0.404	−0.016
Psychological resilience	−0.084	0.333	0.336	0.701	0.360	0.236
Agency	−0.044	0.330	0.404	0.360	0.750	0.270
Family decision-making power	−0.363	0.043	−0.016	0.236	0.270	0.704

**Table 7:** Correlation matrix.

No.	Variable	1	2	3	4	5	6
1.00	Adversity experiences	1.00					
2.00	Reflective selfhood	0.256	1.00				
3.00	Rural woman identity reinterpretation	0.319	0.381	1.00			
4.00	Psychological resilience	−0.084	0.333	0.336	1.00		
5.00	Agency	−0.044	0.330	0.404	0.360	1.00	
6.00	Family decision-making power	−0.363	0.043	−0.016	0.236	0.270	1.00

The focal measurement model showed acceptable fit and outperformed the merged-factor and one-factor alternatives. These results support the distinctiveness of adversity experiences, reflective selfhood, rural woman identity reinterpretation, psychological resilience, agency, and family decision-making power in the present sample. The alternative models fit worse, reducing concern that the results reflect a single undifferentiated response tendency (Table 8). Common method checks also reduced, but did not eliminate, concern about shared response tendencies. Harman’s single-factor test indicated that the first factor accounted for less than 50% of variance, the one-factor CFA model fit substantially worse than the focal model, and the marker-variable robustness model did not materially change the focal moderation result (Table 9). Longitudinal measurement checks supported configural and metric invariance and partial scalar invariance for repeated constructs. Released intercepts are reported transparently in Table 10, and the results should be interpreted as supporting lagged associations rather than strong latent mean comparisons. Nevertheless, the measures should be treated as context sensitive operationalizations requiring further validation.

**Table 8:** Confirmatory factor analysis and alternative model comparisons.

Model	Chi-square	df	Chi-square/df	CFI	TLI	RMSEA	SRMR	AIC	BIC	Interpretation
Six-factor measurement model	1244.07	887	1.40	0.943	0.939	0.031	0.042	199.95	613.86	Acceptable fit
Merged reflective selfhood/social identity model	1624.22	892	1.82	0.883	0.876	0.045	0.052	188.10	581.92	Worse than focal model
One-factor model	4785.81	902	5.31	0.380	0.350	0.102	0.144	152.71	506.35	Poor fit

**Table 9:** Common method bias checks.

Check	Value	Criterion	Decision
Harman single-factor test	17.85	< 50% first-factor variance	Passed
One-factor CFA comparison	One-factor model fit substantially worse than focal model	Inferior fit to focal CFA	Passed
Marker-variable strategy	Unrelated color-preference marker included in robustness model	Focal estimates remain substantively unchanged	Passed

**Table 10:** Longitudinal measurement invariance checks.

Construct	Model	Chi-square	df	CFI	TLI	RMSEA	SRMR	Delta CFI	Delta RMSEA	Released intercepts	Decision
Reflective selfhood	Configural	76.66	53	0.985	0.981	0.031	0.037				Passed
Reflective selfhood	Metric	80.86	58	0.981	0.979	0.033	0.039	−0.004	0.002		Passed
Reflective selfhood	Partial scalar	87.36	62	0.975	0.975	0.037	0.042	−0.006	0.004	RS_T2_3 intercept	Partially passed
Rural woman identity reinterpretation	Configural	78.46	53	0.984	0.980	0.032	0.034				Passed
Rural woman identity reinterpretation	Metric	82.66	58	0.980	0.978	0.034	0.036	−0.004	0.002		Passed
rural woman identity reinterpretation	Partial scalar	89.16	62	0.974	0.974	0.038	0.039	−0.006	0.004	RIR_T2_3 intercept	Partially passed
Psychological resilience	Configural	170.67	169	0.999	0.999	0.005	0.031				Passed
Psychological resilience	Metric	174.87	178	0.995	0.997	0.007	0.033	−0.004	0.002		Passed
Psychological resilience	Partial scalar	181.37	186	0.989	0.993	0.011	0.036	−0.006	0.004	PR_T3_3 intercept	Partially passed
Agency	Configural	48.88	53	1.00	1.00	0.000	0.028				Passed
Agency	Metric	53.08	58	0.999	1.00	0.002	0.030	−0.001	0.002		Passed
Agency	Partial scalar	59.58	62	0.993	0.997	0.006	0.033	−0.006	0.004	AG_T3_3 intercept	Partially passed

**Figure 1:**
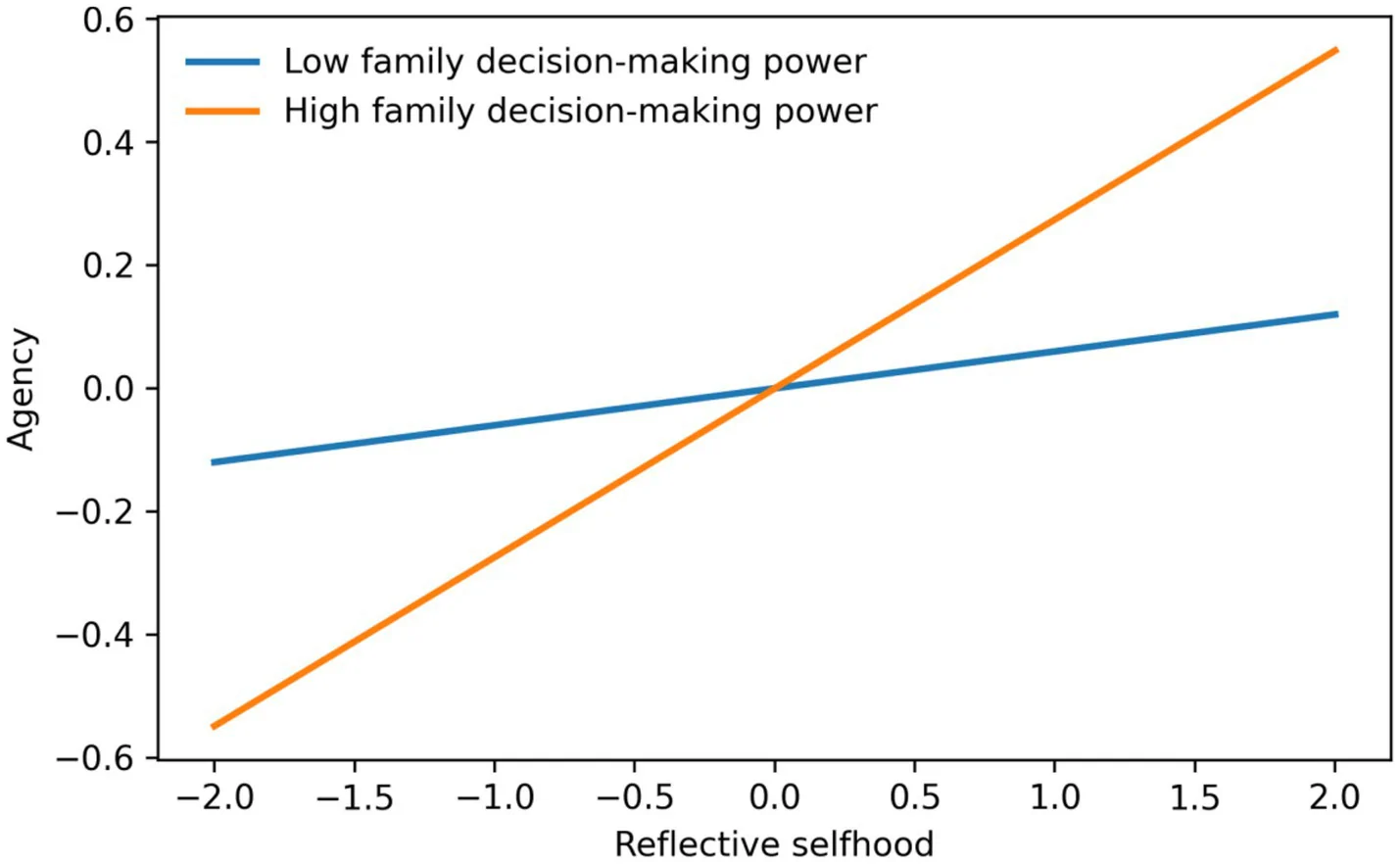
Family decision-making power moderates the association between reflective selfhood and agency.

T1 adversity experiences were positively associated with T2 reflective selfhood and rural woman identity reinterpretation. T2 reflective selfhood and rural woman identity reinterpretation were positively associated with T3 psychological resilience and agency after baseline controls. The direct path from adversity to resilience was negative and statistically significant; the direct path to agency was also negative but weaker and not statistically robust (beta = −0.085, *p* = 0.067). This pattern is consistent with a burden-and-response interpretation in which adversity remains burdensome while reflective and identity based processes are linked to better psychological functioning. The interaction between reflective selfhood and family decision-making power was significant, indicating that reflection was more strongly associated with agency when women had greater voice in family decisions (Table 11; Figure 1).

**Table 11:** Longitudinal path estimates.

Outcome	Predictor	Beta	SE	*p*	95% CI	Result
Reflective selfhood	Adversity experiences	0.219	0.048	<0.001	[0.125, 0.313]	Supported
Rural woman identity reinterpretation	Adversity experiences	0.209	0.054	<0.001	[0.104, 0.314]	Supported
Rural woman identity reinterpretation	Reflective selfhood	0.240	0.044	<0.001	[0.155, 0.326]	Supported
Psychological resilience	Adversity experiences	−0.127	0.047	0.007	[−0.219, −0.036]	Supported
Psychological resilience	Reflective selfhood	0.235	0.047	<0.001	[0.143, 0.327]	Supported
Psychological resilience	rural woman identity reinterpretation	0.210	0.046	<0.001	[0.119, 0.301]	Supported
Agency	Adversity experiences	−0.085	0.047	0.067	[−0.177, 0.006]	Not supported
Agency	Reflective selfhood	0.167	0.046	<0.001	[0.077, 0.257]	Supported
Agency	rural woman identity reinterpretation	0.321	0.043	<0.001	[0.237, 0.405]	Supported
Agency	Family decision-making power	0.195	0.047	<0.001	[0.103, 0.286]	Supported
Agency	Reflective selfhood x family decision-making power	0.107	0.039	0.006	[0.031, 0.183]	Supported

Indirect association analyses showed positive prospective statistical patterns through T2 reflective selfhood and T2 rural woman identity reinterpretation (Table 12). These results are consistent with an intervening process interpretation, but they do not establish a directional process because unmeasured third variables and reciprocal processes remain possible. Exploratory moderation results indicated that reflective selfhood was positively associated with agency at both high and low levels of family decision-making power, with a stronger association when family decision-making power was high (Table 13). Because *post hoc* power for the interaction was approximately 0.40, this moderation pattern should be interpreted cautiously and requires replication in larger samples. Robustness checks showed that the interaction pattern remained substantively similar across demographic adjusted, marker adjusted, and IPW adjusted specifications (Table 3).

**Table 12:** Bootstrapped indirect associations.

Indirect path	Effect	Boot SE	95% CI lower	95% CI upper	Result
ADV → RS → PR	0.052	0.015	0.025	0.085	Supported
ADV → RS → AG	0.037	0.013	0.014	0.065	Supported
ADV → RIR → PR	0.044	0.015	0.018	0.076	Supported
ADV → RIR → AG	0.067	0.019	0.032	0.107	Supported
ADV → RS → RIR → PR	0.011	0.004	0.005	0.020	Supported
ADV → RS → RIR → AG	0.017	0.005	0.008	0.029	Supported

**Table 13:** Simple slopes for reflective selfhood predicting agency.

Family decision-making power	Simple slope RS → Agency	Interpretation
Low (−1 SD)	0.060	Positive but weaker
High (+1 SD)	0.274	Stronger positive association

## 5 Discussion

### 5.1 Main findings

This study examined how rural women’s adversity experiences are prospectively associated with resilience and agency through reflective selfhood and rural woman identity reinterpretation. The findings suggest that adversity should not be understood only as psychological risk, although risk remains central. The psychological significance of adversity lies not in hardship itself, but in how women may narratively and reflectively organize lived constraint into self-understanding and possible action. Adversity remains a structural burden, while reflective and identity based processes represent interpretive responses that may develop under constraint. The positive association between adversity and reflective selfhood should not be read as romanticizing hardship or as evidence that hardship causes growth. Rather, it suggests that repeated exposure to family responsibility, limited opportunity, and low recognition may be associated with efforts to understand who one is, why life has taken a certain shape, and what forms of action remain available. Reflection may function as one way of organizing experience. It does not remove structural constraint, but it may help women distinguish personal failure from social limitation. The nonsignificant direct association between adversity and agency also has theoretical meaning. The estimate was small and negative (beta = −0.085, *p* = 0.067), which is consistent with the claim that adversity does not automatically become agency. Instead, agency appears more closely linked to interpretive and identity based processes. At the same time, the analytic sample had limited power to detect a very small direct effect of beta around 0.08 (approximate power = 0.37), so the absence of a significant direct path should not be over interpreted. Overall, the results support a cautious prospective interpretation rather than a claim that one psychological factor definitively causes another.

### 5.2 Reflective selfhood as a context-sensitive construct

Reflective selfhood was associated with both resilience and agency, but this result should be interpreted as a lagged association rather than as proof of a causal psychological mechanism. The pattern supports the idea that self-understanding is not merely cognitive. It may help organize fragmented hardship into a more coherent account of self and possibility. In rural contexts, reflection may help women connect sacrifice, responsibility, constraint, and aspiration into a more coherent self-narrative. This coherence may be one reason reflective selfhood is linked to psychological resilience. At the same time, alternative explanations remain plausible: women with stronger baseline psychological resources, more supportive families, or greater community recognition may be more likely both to report reflective selfhood and to report later resilience or agency. The construct also requires careful boundary setting. Reflective selfhood is not equivalent to self-efficacy, because it does not primarily ask whether women believe they can perform a task. It is not equivalent to resilience, because it is not the capacity to recover from difficulty. It is not simply self-reflection, because the focus is not introspective frequency but interpretation of life experience under social constraint. Nor is it rumination, because it emphasizes meaning making and future orientation rather than repetitive distress. This boundary matters for Q1-level theorizing: the contribution is not another positive self-belief variable, but a context sensitive process through which women interpret the relationship between personal biography and social structure (Gecas, 2003; Ryff, 2014).

### 5.3 Rural woman identity reinterpretation and narrative revaluation

Rural woman identity reinterpretation also mattered, although the present measure should be interpreted as an initial operationalization of identity reinterpretation rather than a complete identity development scale. Rural women’s identities are often shaped by deficit narratives that associate rural womanhood with dependence, limited education, silence, or domestic obligation. The findings are consistent with a narrative revaluation process in which rural womanhood can be re described as dignity, contribution, care knowledge, competence, and community continuity (Fricker, 2007; Stryker and Burke, 2000).

This identity argument is deliberately not a celebration of hardship. A deficit frame sees rural womanhood mainly as deprivation; a romantic frame treats endurance as moral virtue. Both are incomplete. Rural woman identity reinterpretation occupies a more careful position. It suggests that women may reinterpret a devalued social identity without denying the inequalities that make such reinterpretation necessary.

### 5.4 Family decision-making power as relational space for agency

Family decision-making power was examined as a contextual condition, but the evidence should be treated as preliminary. Exploratory moderation results suggested that reflective selfhood was more strongly associated with agency when women reported more voice in household decisions. This pattern is theoretically consistent with the idea that agency is not supported by psychological reflection alone; it also requires relational and structural space for action. However, the modest interaction and limited statistical power mean that this finding should not be treated as one of the strongest conclusions of the article (Kabeer, 2020; Thoits, 2011).

The moderation effect was statistically reliable but modest in size. It should not be read as showing that household voice fully determines women’s agency. It provides preliminary evidence that reflective self-understanding may have more room to become action when women can participate in decisions about money, children’s education, health care, work, mobility, and family responsibilities. This interpretation protects the study from an overly individualistic empowerment narrative, but the pattern requires replication in larger samples.

### 5.5 Theoretical implications

The study proposes and preliminarily tests a narrative informed, context sensitive model of rural women’s selfhood and agency under structural constraint. Its theoretical contribution is best understood as opening a conceptual pathway rather than confirming a mature theory. The findings suggest that adversity remains a structural burden, while reflective selfhood and rural woman identity reinterpretation may function as narrative informed interpretive processes through which women organize lived constraint, dignity, and possible action.

The focal contribution lies in the theoretical positioning of reflective selfhood and rural woman identity reinterpretation. Reflective selfhood is treated as a preliminary measurable proxy for author level self-interpretation. Rural woman identity reinterpretation is treated as a preliminary measure of narrative revaluation of a socially devalued identity. The exploratory moderation finding suggests that household decision space may shape whether reflective selfhood is associated with agency, but this pattern requires replication because statistical power for the interaction was limited.

### 5.6 Practical implications

The findings suggest possible directions for program design, but they do not provide direct intervention evidence. Rural development programs may consider creating reflective and identity recognition spaces in which women can name constraints, connect personal experience with shared social patterns, and discuss rural womanhood as dignity, care knowledge, contribution, and competence. Future intervention research should test whether these practices actually affect agency, resilience, or wellbeing (UN Women, 2023, 2024).

These implications should remain tied to material and institutional support. Reflection based programs should not ask women to reinterpret hardship while unequal labor, limited services, and household exclusion remain unchanged. Family decision making education should also involve spouses, older family members, community workers, and local institutions that shape whether women’s voices are heard (Wang and Tang, 2020; Jacka, 2012).

### 5.7 Alternative explanations, measurement caution, and guardrails

Several alternative explanations deserve attention. Reflective selfhood may partly capture a generally positive personality orientation, and women with stronger community support, more secure household resources, higher literacy, or more recognized family roles may be better positioned both to reflect on their experiences and to report higher later agency. Reverse direction is also possible. Women who already feel more agentic may be more willing to interpret their lives reflectively, name constraints, or reinterpret rural womanhood as a source of dignity. Although the three-wave design reduces some concerns relative to same-wave models, it does not establish directionality or rule out reciprocal processes.

Measurement caution is also necessary because all focal variables were assessed through self-report questionnaires. The measurement and common method checks reduced concern that the findings simply reflect one undifferentiated response tendency, but they do not eliminate possible shared method variance, social desirability, acquiescent responding, or culturally patterned ways of describing hardship and endurance. This is especially important because reflective selfhood, identity reinterpretation, resilience, and agency are conceptually related self-interpretive constructs. Some of the observed associations may therefore partly reflect common response style or shared self-narrative rather than fully distinct psychological processes.

Reflective selfhood and rural woman identity reinterpretation have theoretical value because they propose narrative-informed constructs for studying rural women’s lives, but the present evidence remains preliminary. The sample supports internal consistency, discriminant validity, and preliminary external evidence, but it does not demonstrate stable equivalence across regions, ethnic groups, age groups, family structures, or cultural contexts. Several other constructs also showed AVE values near 0.50, indicating moderate rather than strong convergent validity. Future research should therefore use independent samples, cross-regional tests, qualitative interviews, mixed-method validation, behavioral or family-reported indicators where feasible, and more extensive scale-development procedures.

These guardrails are important because the paper should not shift responsibility for inequality onto individual women. Reflective selfhood is not a substitute for income security, public services, legal protection, child-care support, or fair recognition of labor. Rural woman identity reinterpretation should not be used to suggest that women can overcome structural disadvantage simply by changing their self-narrative. The model is best read as showing how interpretive psychological processes may coexist with, and remain constrained by, unequal material and relational conditions. In this sense, the study does not present reflection or identity reinterpretation as solutions to structural inequality. Rather, it suggests that women’s interpretive processes can be psychologically meaningful while still being shaped by the broader social, economic, family, and cultural conditions that limit or enable agency. Future research should further examine how culturally specific meanings of womanhood, identity, resilience, and agency shape these interpretive processes across different rural, ethnic, and national contexts. Comparative studies across diverse cultural settings would help clarify which aspects of the proposed model reflect broader psychological processes and which are embedded within particular social structures (McAdams and McLean, 2013; Adler et al., 2016).

### 5.8 Limitations and future research

Several limitations should be noted. First, the three-wave design improves temporal ordering, but it cannot establish causality, fully separate reciprocal processes, or rule out unmeasured third variables. Reflective selfhood may be associated with later agency, but women who already feel more agentic may also become more reflective about their life conditions. The moderation result involving family decision-making power should also be treated as preliminary and exploratory because *post hoc* power for the interaction was only about 0.40.

Second, the study relied on self-report measures. Although CFA comparisons, HTMT evidence, a marker-variable check, and common method diagnostics were used, these tests are diagnostic rather than definitive. They cannot show that common method bias was absent. Future studies should combine survey data with qualitative narratives, family or community reports, behavioral indicators of decision participation, and longitudinal designs capable of testing reciprocal effects.

Third, attrition was not completely random. Women who dropped out by T3 reported significantly lower baseline psychological resilience and agency, and they also tended to report higher adversity and lower family decision-making power. This means that the final analytic sample may underrepresent women facing the greatest psychological and relational challenges. The IPW robustness analysis helps assess sensitivity to selective retention, but it cannot correct for unobserved reasons for dropout.

Fourth, measurement limitations remain important. AVE values near or below 0.50 for adversity experiences, psychological resilience, and family decision-making power indicate moderate rather than strong convergent validity for some constructs. Reflective selfhood and rural woman identity reinterpretation are preliminary, self-developed, context-sensitive measures, and they require further validation across regions, ethnic groups, age cohorts, family structures, and cultural settings before they can be treated as established scales.

Fifth, although the study is narrative-informed, it does not directly analyze life stories, interview narratives, or discourse data. It therefore cannot show how rural women narrate adversity in their own words, how they sequence life events, or how they construct meaning through actual storytelling. Future research should combine the present longitudinal survey model with life-history interviews, narrative analysis, participatory storytelling, and family-level qualitative data. Such work would strengthen both the measurement foundation and the narrative psychological interpretation of the model, and would also place the findings more clearly within wider literatures on women, identity, resilience, agency, and culturally situated development (Pals, 2006; Hammack, 2008).

## 6 Conclusion

This study provides preliminary narrative-informed longitudinal evidence on rural women’s selfhood and agency under structural constraint. The findings are consistent with the view that reflective selfhood and rural woman identity reinterpretation may function as interpretive processes through which women organize adversity, dignity, and possible action. However, the evidence should be interpreted as self-report-based prospective associations rather than proof of causal psychological mechanisms. Adversity remains a burden and should not be romanticized as inherently beneficial. Both constructs remain preliminary and require further validation across independent samples and diverse rural and cultural contexts. The exploratory moderation finding concerning family decision-making power also requires replication in larger samples. The study should therefore be read as an initial step toward a more narrative-informed and context-sensitive psychology of rural women’s agency, rather than as a definitive account of how resilience develops under hardship.

## Data Availability

The raw data supporting the conclusions of this article will be made available by the authors, without undue reservation.
